# Koreans Invested in Making Caregivers Health Important (KIMCHI): A National Community‐based Project to Enhance Knowledge, Attitudes, and Behaviors about Advanced Planning and Directives for the Korean American Community

**DOI:** 10.1002/alz70858_103256

**Published:** 2025-12-26

**Authors:** Bora Nam, Daren Huang, Hye‐Won Shin, Eun Jeong Lee, Nicole Phan, Stacy W Yun, Van Ta Park

**Affiliations:** ^1^ University of California San Francisco School of Nursing, San Francisco, CA, USA; ^2^ Somang Society, Cypress, CA, USA; ^3^ The UC Irvine Institute for Memory Impairments and Neurological Disorders, Irvine, CA, USA; ^4^ AARIN, Wood‐Ridge, NJ, USA; ^5^ NYC College of Technology at City University of New York (CUNY), Brooklyn, NY, USA; ^6^ Peninsula Vet Center, Menlo Park, CA, USA; ^7^ Asian American Research Center on Health (ARCH), University of California San Francisco, San Francisco, CA, USA

## Abstract

**Background:**

Older Korean adults traditionally engage in indirect communication and are uncomfortable with topics such as advance care planning and treatment options for health conditions such as Alzheimer's disease and related dementias (ADRD). The overarching goal of a national, culturally tailored, bilingual (English, Korean) community engagement project called, Koreans Invested in Making Caregivers Health Important (KIMCHI), is to educate and promote community discussions on aging and caregiving topics, such as advanced care planning/directives among older Korean Americans with ADRD and their caregivers.

**Method:**

Seven in‐person workshops were hosted in collaboration with two community organizations that serve Korean Americans: Somang Society and Asian American Resource and Information Network, Inc. Pre‐ and post‐workshop assessments measured knowledge (10 items), attitudes (7 items), and behaviors (6 items) on advance directives, where participants answered on an “agree‐disagree‐neutral” scale. Paired‐sample t‐tests were conducted to assess mean (M) changes. Participants also completed a satisfaction survey to evaluate the workshop.

**Result:**

There were 204 participants (aged 25‐92 years; M=67; SD=11.4) who were mostly female (75%) and foreign‐born (98%) and who reported limited English proficiency (87%). Nearly a third (32.4%) were ADRD caregivers or knew or worked with someone who has ADRD. Post‐test results showed significant changes in attitudes (pre: M=10.29; post: M=9.76; t(203)=0.53, *p* < .05) and behaviors (pre: M=9.79; post: M=10.71; t(203)=0.92, *p* < .05) regarding advanced planning and directives. Knowledge scores showed no significant change (pre: M=12.62; post: M=12.50; t(203)=0.36, *p* = .47), though the knowledge pre‐test score was notably high. High satisfaction was reported, with 96.1% of participants expressing overall satisfaction, 90.7% learned something new about advanced directives, 94.1% found the presentations culturally relevant and applicable, and 76% expressed interest in learning more about ADRD.

**Conclusion:**

Findings demonstrate the potential of culturally tailored, community‐based interventions to improve Korean Americans’ attitudes and behaviors toward understanding and engaging in advanced care planning, including making informed decisions about end‐of‐life care. Future studies should further tailor content for diverse learning needs, including real‐life case examples, and ensure more representation in research studies for the Korean American community.

